# Are current chronic fatigue syndrome criteria diagnosing different disease phenotypes?

**DOI:** 10.1371/journal.pone.0186885

**Published:** 2017-10-20

**Authors:** Laura Maclachlan, Stuart Watson, Peter Gallagher, Andreas Finkelmeyer, Leonard A. Jason, Madison Sunnquist, Julia L. Newton

**Affiliations:** 1 Institute of Cellular Medicine, Newcastle University, Newcastle upon Tyne, United Kingdom; 2 Institute of Neuroscience, Newcastle University, Newcastle upon Tyne, United Kingdom; 3 Northumberland Tyne and Wear NHS Foundation Trust, Newcastle upon Tyne, United Kingdom; 4 DePaul University, Chicago, IL, United States of America; 5 Newcastle Hospitals NHS Foundation Trust, Newcastle upon Tyne, United Kingdom; Universitat Wien, AUSTRIA

## Abstract

**Importance:**

Chronic fatigue syndrome (CFS) is characterised by a constellation of symptoms diagnosed with a number of different polythetic criteria. Heterogeneity across these diagnostic criteria is likely to be confounding research into the as-yet-unknown pathophysiology underlying this stigmatised and debilitating condition and may diagnose a disease spectrum with significant implications for clinical management. No studies to date have objectively investigated this possibility using a validated measure of CFS symptoms–the DePaul Symptom Questionnaire (DSQ).

**Objective:**

To examine whether current CFS diagnostic criteria are identifying different disease phenotypes using the DSQ.

**Design:**

Case control study.

**Setting:**

Clinical Research Facility of the Royal Victoria Infirmary, Newcastle upon Tyne, UK.

**Participants:**

49 CFS subjects and ten matched, sedentary community controls, excluded for co-morbid depression.

**Main outcomes and measures:**

Self-reported autonomic and cognitive features were assessed with the Composite Autonomic Symptom Score (COMPASS) and Cognitive Failures Questionnaire (COGFAIL) respectively. Objective autonomic cardiovascular parameters were examined using the Task Force® Monitor and a battery of neuropsychological tests administered for objective cognitive assessment.

**Results:**

Self-reported autonomic and cognitive symptoms were significantly greater in CFS subjects compared to controls. There were no statistically significant differences in objective autonomic measures between CFS and controls. There were clinically significant differences between DSQ subgroups on objective autonomic testing. Visuospatial memory, verbal memory and psychomotor speed were significantly different between DSQ subgroups.

**Conclusions and relevance:**

The finding of no significant differences in objective autonomic testing between CFS and control subjects may reflect the inclusion of sedentary controls or exclusion for co-morbid depression. Consistent exclusion criteria would enable better delineation of these two conditions and their presenting symptoms. Findings across CFS subgroups suggest subjects have a different disease burden on subjective and objective measures of function, autonomic parameters and cognitive impairment when categorised using the DSQ. Different CFS criteria may at best be diagnosing a spectrum of disease severities and at worst different CFS phenotypes or even different diseases. This complicates research and disease management and may contribute to the significant stigma associated with the condition.

## Introduction

Chronic fatigue syndrome (CFS) is a disabling and stigmatising illness of unknown aetiology. Its current UK prevalence is estimated to be at least 0.2–0.4%[1]; studies using broader screening procedures have yielded prevalence estimates of over 2%[2]. CFS is a diagnosis of exclusion based on a number of different sets of polythetic diagnostic criteria developed empirically over the last three decades to reflect the many presenting symptoms. The diagnostic and prognostic salience of the different signs and symptoms is unknown and this inclusion of multiple symptoms involving many body systems complicates clinical diagnosis and is likely to add to the stigma associated with the condition.

It is widely recognised that there are limitations to these diagnostic sets and that there is disparity between them[3–7]. The different sets may identify different categorical groups differing in phenotypic pattern and which can be conceptualised as subtypes of CFS or even different disorders or may relate to a continuum across which different thresholds are arbitrarily met dependent on diagnostic schedule used[8, 9].

The DePaul Symptom Questionnaire (DSQ) was devised by Leonard Jason *et al* as a development of the CFS Questionnaire[10] to better, and more consistently, assess the “core” symptoms of CFS. Frequency and severity of symptoms, demographics, and medical, occupational, and social history are self-reported over a retrospective six-month period using a 5-point Likert scale to give a “diagnosis” of CFS based on the Fukuda criteria[11], the 2003 Canadian clinical[12] or research[13] criteria and the 2011 Canadian consensus criteria [14] (Table 1). It has been shown to have good inter-rater and test-retest reliability and validity in the differentiation of individuals with CFS, major depressive disorder (MDD), and healthy controls[15–17].

**Table 1:** Chronic fatigue syndrome diagnostic criteria.

Criteria	Major	Minor
**Fukuda criteria/ CDC 1994 criteria [11]**	• ≥ six months of severe chronic fatigue not due to exertion or other illness and interfering with daily life	≥ four of the following eight symptoms: • Post-exertional malaise of >24 hours • Unrefreshing sleep • Impairment to short-term memory or concentration • Myalgia • Arthralgia without swelling/erythema • Headaches of new type • Lymphadenopathy • Frequent or recurring sore throat
**Canadian consensus 2003 [12]** **(DSQ 2003 clinical guidelines)**	≥ six months of: • Significant new-onset, unexplained, persistent fatigue that reduces activity level, and/or • Post-exertional malaise, and/or • Sleep dysfunction and/or • Pain in the form of widespread myalgia/arthralgia or headaches	≥ two neurological/cognitive symptoms: • Confusion • Poor concentration and short-term memory • Poor information processing, categorising and word retrieval • Perceptual and sensory disturbances • Ataxia • Muscle weakness • Fasciculations and ≥ one from two of the following categories: • Autonomic: orthostatic intolerance, PoTS, nausea, irritable bowel, urinary frequency, palpitations, exertional dyspnoea • Neuroendocrine: loss of thermostatic stability, weight change • Immune: lymphadenopathy, recurrent sore throat, general malaise, sensitivities to food/medications
**DSQ revised Canadian research criteria [18]**	• New, persistent or recurring fatigue for at least six months which impacts usual activities • Post-exertional malaise and/or post-exertional fatigue • Unrefreshing or disturbed sleep • Widespread or migratory pain • ≥ two neurological or cognitive symptoms • ≥ one autonomic/neuroendocrine/immune manifestation	Absence of any active medical condition that may explain the presence of chronic fatigue
**Canadian (International) consensus 2011 [14]**	• Postexertional neuroimmune exhaustion with marked, prolonged post-exertional physical or cognitive fatigue with extended recovery period	≥ one symptom from three of these categories: • Neurocognitive impairment • Pain • Sleep disturbance • Neurosensory, perceptual and motor disturbances ≥ one symptom from three of these categories: • Flu-like symptoms • Susceptibility to viral infections • Gastro-intestinal tract symptoms • Genitourinary symptoms • Sensitivities ≥ one symptom from these categories: • Cardiovascular • Respiratory • Loss of thermostatic stability • Intolerance of extremes of temperature

Autonomic dysfunction in CFS has consistently been reported in studies[19], as has a potential sequela cognitive dysfunction [20]. However, the nature of these finding lacks uniformity, which may be related to heterogeneity within CFS patients or to systematic differences in sample selection between studies based on differing inclusion criteria, diagnostic rigour or recruitment strategy. Here we report subjective and objective measures of disease severity, autonomic dysfunction and cognitive impairment in CFS subjects and matched comparators, as well as the impact of DSQ categorisation between subgroups, to elicit whether different diagnostic criteria identify a spectrum of disease phenotypes.

## Materials and methods

### Recruitment

Participants were recruited as part of a Medical Research Council funded study Understanding the pathogenesis of autonomic dysfunction in chronic fatigue syndrome and its relationship with cognitive impairment. Recruitment was conducted between November 2012 and April 2014. CFS subjects were recruited from the Newcastle upon Tyne and regional specialist CFS services. All met Fukuda criteria as diagnosed by physician via clinical interview and confirmed with administration of the DSQ during study screening. Matched sedentary controls were recruited via university volunteer databases, advertisements and word of mouth. Participants with co-morbid hypertension or psychiatric disorder diagnosed using the Structured Clinical Interview for DSM-IV Research Version were excluded. Cardioactive medications were withheld for 72 hours prior to assessment. Patients and controls were matched for age, gender, employment or education status and premorbid IQ (using the validated National Adult Reading Test.

The MRC study was conducted in accordance with the recommendations for physicians involved in research on human subjects adopted by the 18th World Medical Assembly, Helsinki 1964 and later revisions. Informed consent was obtained.

Favourable ethical opinion was obtained from the NRES Committee North East—Newcastle & North Tyneside 2 prior to commencement of the study. Local research and development approval was obtained from Newcastle upon Tyne Hospitals NHS Foundation Trust’s Research and Development office. All participants provided written, informed consent.

### DePaul symptom questionnaire

The DSQ is a self-reported 99-item questionnaire measuring CFS symptoms, demographic information and medical, social and occupational history according to the Canadian Clinical Criteria, the ME International Consensus Criteria, and the Fukuda criteria[16, 21]. It is available in the shared library of Research Electronic Data Capture (REDCap), hosted at DePaul University: https://redcap.is.depaul.edu/surveys/?s=tRxytSPVVw. After scoring for symptom frequency and severity, patients are categorised into one of four criteria groups: the Fukuda; the Fukuda+2003 Canadian Clinical; the Fukuda+2003 Canadian Research, or the Fukuda+2003+2011 Canadian (Table 1). Those meeting the Fukuda+2003 Clinical differ from the Fukuda+2003 Research in that they do not meet the more stringent Research criteria requiring the presence of major symptoms.

### Other questionnaires

The Fatigue Impact Scale (FIS) assesses the impact that subjective fatigue has on daily functioning[22]. Forty items are scored on a 5-point Likert scale providing a continuous scale of 0–160. A higher score indicates greater impact.

Participants completed the Autonomic Symptom Profile[23] as a self-report measure of autonomic symptoms. However, scoring was performed according to the recently abbreviated and psychometrically improved version of this questionnaire, the Composite Autonomic Symptom Score 31 (COMPASS 31)[24]. Scoring consists of 31 items from six domains–orthostatic intolerance, vasomotor, secretomotor, gastrointestinal, bladder, pupillomotor–each weighted according to number of items and clinical relevance. Weighted individual domain scores are totalled to a maximum of 100, indicating greater symptom load.

The Cognitive Failures Questionnaire (COGFAIL)[25] comprises 25 questions measuring the frequency of self-reported failures in perception, memory and motor function in the previous six months and is answered on a 5-point Likert scale giving a possible total score of 100. Higher scores indicate greater impairment.

There is currently no standardised way to measure self-reported activity levels. The short version of the International Physical Activity Questionnaire (IPAQ) gives a measure of physical activity over the preceding seven days[26] and has been shown to be a reliable measure of self-reported physical activity[27]. The questionnaire asks about three types of activity: walking, moderate-intensity activities and vigorous-intensity activities, undertaken during leisure time, domestic and gardening activities, work-related physical activity and transport-related physical activity. The duration (minutes) and frequency (days) of activity is scored to give METs, multiples of resting metabolic rate, equated to levels of activity and described as low, moderate or high.

### Participant characteristics

Demographic information, symptom burden, function and diagnostic information were obtained by participant report and questionnaire, as above.

### Autonomic assessment

Autonomic assessment was conducted at the Clinical Research Facility at the Royal Victoria Infirmary. The Task Force® Monitor (TFM, CNSystems Medizintechnik, Graz, Austria) was used to record and analyse continuous heart rate (HR) (electrocardiogram (ECG)) and beat-to-beat blood pressure (BP) assessment. It is a reliable and reproducible method of non-invasive autonomic assessment[28, 29].

Participants were instructed to eat a light breakfast, avoid caffeine and alcohol on the day of testing and refrain from nicotine for two hours before assessment. All assessments were performed between 9–10am. TFM recordings were taken during a ten-minute supine rest, a two-minute active stand and a Valsalva manoeuvre.

### Cognitive assessment

The battery of neuropsychological tests focussed on memory and concentration, shown to be features of cognitive impairment in CFS[30], and assessed verbal and non-verbal memory, recall, recognition and learning skills. Tests reported here are the Rey Auditory-verbal learning test (AVLT) to assess verbal learning and memory, the Digit Symbol Substitution Test (DSST) to assess psychomotor speed, Digit Span for working and short-term memory, Spatial Span for working and short-term dynamic visuospatial memory and the Visual Patterns Test (VPT) to assess short-term fixed visuospatial memory. Tests were conducted under time pressure and commenced immediately after autonomic assessment. Caffeine, alcohol and nicotine were avoided as above.

### Statistical analysis

Data were analysed using GraphPad Prism version 6. The difference between CFS patients and control participants in normally-distributed continuous data was analysed using the unpaired t-test, at a 5% and 1% significance level, as indicated. Unpaired continuous skewed data were analysed using the non-parametric Mann Whitney U test. The differences between the four patient sub-groups according to DSQ criteria was analysed using ANOVA or the Kruskal-Wallis test for non-parametric datasets. A 5% significance level is given. For significant ANOVA findings Bonferroni-corrected post-hoc test results are reported. (In addition, as increasingly stricter criteria are applied to the four DSQ diagnostic groups, the assigned sub-group membership can be considered an ordinal variable. Accordingly, Kendall’s tau-b tests were performed to assess the strength of the monotonic relationship between diagnostic category and the various measures.)

## Results

### Study cohort

Seventy-six potential participants were invited for screening, of whom sixty-nine were eligible. Eight withdrew from the study; one was lost to follow-up and one commenced a new medication resulting in loss of eligibility. In total, 59 participants (CFS n = 49; controls n = 10) underwent autonomic and cognitive testing.

### Participant characteristics

Patients and controls were well-matched with no significant differences between age, gender, employment or education status, or pre-morbid IQ (Table 2). Age range was of CFS participants was 23–68 years. Age range of controls was 25–65 years. Co-morbidities were self-reported by affected system and include cardiorespiratory, neurological, gastrointestinal and endocrine disease. The presence of co-morbidities was higher in the CFS group compared to controls (92% versus 70%). Among CFS participants, those meeting the Fukuda+2003 Research and Fukuda+2003+2011 criteria had lower educational attainment and were less likely to be in employment.

**Table 2:** Baseline characteristics.

		Controls	Fukuda	Fukuda	Fukuda +	Fukuda
		(n = 10)	alone	+ 2003	2003	+ 2003
			(n = 6)	Clinical	Research	+ 2011
				(n = 9)	(n = 8)	(n = 26)
**Age (years)**						
mean (SD)		49 (15.3)	43 (14.5)	50 (14.0)	41 (10.9)	47 (11.1)
range				26–68	23–61	
**Male** n (%)		3 (30%)	1 (17%)	5 (56%)	1 (13%)	6 (30%)
**Employed** n (%)	Yes	3 (30%)	3 (50%)	4 (44%)	3 (38%)	7 (27%)
	*Full time*	*1 (10%)*	0 (0%)	4 (44%)	0 (0%)	3 (12%)
	*Part time*	*2 (20%)*	3 (50%)	0 (0%)	3 (38%)	4 (15%)
	*Paid*	*3 (30%)*	3 (50%)	4 (44%)	3 (38%)	7 (27%)
	*Voluntary*	*0 (0%)*	0 (0%)	0 (0%)	0 (0%)	0 (0%)
**Student** n (%)		2 (20%)	0 (0%)	0(0%)	0(0%)	0(%)
**Years in Education** n (%)	≤11	1 (10%)	1 (17%)	1 (11%)	0 (0%)	8 (31%)
	>11	9 (90%)	5 (83%)	8 (89%)	8 (100%)	18 (69%)
**Highest qualification** n (%)	None	0 (0%)	0 (0%)	0 (0%)	1 (13%)	2 (8%)
	GCSE	2 (20%)	1 (17%)	1 (11%)	0 (0%)	11 (42%)
	A level	2 (20%)	0 (0%)	1 (11%)	3 (38%)	6 (23%)
	Degree	6 (60%)	5 (83%)	7 (78%)	4 (50%)	7 (27%)
**Premorbid IQ** mean (SD)		122 (4.3)	119 (6.6)	117 (6.6)	123 (3.1)	116 (9.2)
**Co- morbidities** n (%)		7 (70%)	5 (83%)	8 (89%)	7 (88%)	25 (96%)
**Cardioactive medication** n (%)		0 (0%)	1 (17%)	5 (56%)	2 (25%)	10 (38%)

### Symptom burden

CFS participants self-reported statistically significantly greater fatigue, cognitive impairment and autonomic symptoms (Table 3). IPAQ scores in both groups were lower than those seen in active, healthy volunteers in other studies, who have shown median scores of 4500–6000[31, 32], and reflect the sedentary lifestyle of participants in this study. Nevertheless, controls had significantly higher IPAQ scores than CFS subjects (1230 (IQR 821) v. 203 (1002)) which may represent the cumulative effect of nonstrenuous activity not achievable by CFS patients because of illness burden.

**Table 3:** IPAQ scoring and outcome measures by DSQ.

	Controls	all CFS	Fuk-	Fukuda	Fukuda	Fukuda +	p value	p
			uda	+ 2003	+ 2003	2003 +	(ANOVA/	value
			alone	Clinical	Re-	2011	Mann	(Kend-
					search		Whitney)	all tau-
	(n = 10/	(n = 49/	(n = 6)	(n = 9/	(n = 8/	(n = 26/		b)
	9#)	46#)		8#)	7#)	25#)		
**FIS**	n/a	91.7	58.2^$^	81.7	93.1	102.5^$^	0.013*	0.004
		(32.5)	(34.1)	(19.3)	(22.4)	(33.3)		**
**COGFAIL**	32.3	53.9	42.0	49.6	54.1	58.2	0.250	0.094
	(10.0)	(19.1)**	(17.4)	(15.8)	(20.9)	(19.3)		
**COM-**	8.1	38.6	26.3^$^	31.3	37.2	44.4^$^	0.015*	0.002
**PASS 31**	(5.7)	(15.2)**	(13.5)	(12.8)	(6.7)	(15.8)		**
**IPAQ**							
**score**							
median	1230	203	819	908	208	33	0.041*
(IQR)	(821)	(1002)*	(1418)	(983)	(1040)	(565)	
Low (n)	2	29	2	4	4	19	
	(22%)	(63%)	(33%)	(50%)	(57%)	(76%)	
Mod-	6	16	4	4	3	5	
erate (n)	(67%)	(35%)	(67%)	(50%)	(43%)	(19%)	
High (n)	1 (11%)	1 (2%)	0 (0%)	0 (0%)	0 (0%)	1 (4%)	

# Questionnaire data were incomplete for one or more participants in each of these groups

* significant at 5% level

** significant at 1% level

$ significant Bonferroni-corrected post-hoc tests between these groups

Values expressed as mean (SD)

Across DSQ subgroups there are statistically significant differences in FIS and COMPASS 31 scores, and there was a statistical trend for a monotonic increase of self-reported cognitive impairment with increased DSQ category. Participants meeting the Fukuda+2003+2011 criteria had the highest subjective levels of fatigue, cognitive impairment and symptoms of dysautonomia compared to the other CFS diagnostic subgroups. The lowest scores are seen in the group meeting Fukuda alone.

### Autonomic nervous system at rest

Objective testing did not reveal any statistically significant differences between CFS and control subjects (Table 4). Resting heart rate (HR) and blood pressure (BP) were at the lower end of the normal clinical reference range in both groups. Examination of HR and BP variability appeared to show a shift to increased sympathetic modulation in both groups compared to normal reference ranges. Baroreflex control was within normal limits in CFS and control subjects.

**Table 4:** Autonomic function (heart rate and blood pressure variability and beat statistics, active stand and Valsalva) by DSQ.

		Control	all CFS	Fukuda	Fukuda + 2003	Fukuda + 2003	Fukuda + 2003	p value	p value
					Clinical	Research	+ 2011	(ANOVA/	(Kendall
								Pearson	tau-b)
		(n = 10/9#)	(n = 49/47#)	(n = 6)	(n = 9)	(n = 8)	(n = 26/24#)	χ^2^)	
**Beat statistics**	HR (bpm)	76.62 (7.11)	75.02 (9.84)	70.26 (5.46)	73.07 (9.24)	75.92 (6.36)	76.52 (11.48)	0.501	0.227
	sBP (mmHg)	104.87 (11.19)	108.09 (19.62)	102.94 (20.80)	122.27 (24.99)	100.07 (12.72)	106.83 (17.51)	0.083	0.664
	dBP (mmHg)	67.86 (6.55)	68.81 (11.77)	69.10 (20.49)	71.90 (14.75)	63.82 (6.10)	69.20 (9.50)	0.567	0.821
	mBP (mmHg)	78.23 (7.63)	79.03 (13.06)	78.44 (20.71)	83.65 (15.55)	73.75 (7.06)	79.20 (11.57)	0.497	0.806
**Heart rate variability**	LFnu-RRI	62.42 (12.89)	58.80 (17.08)	55.63 (13.22)	68.60 (13.42)	64.74 (19.54)	54.31 (17.06)	0.111	0.108
	HFnu-RRI	37.58 (12.89)	41.20 (17.08)	44.37 (13.22)	31.40 (13.42)	35.27 (19.54)	45.69 (17.06)	0.111	0.108
	LF/HF-RRI	2.25 (1.16)	2.44 (2.68)	2.23 (1.94)	3.14 (1.87)	4.08 (5.37)	1.75 (1.50)	0.144	0.064
**Diastolic BP variability**	LFnu-dBP	46.81 (19.01)	53.63 (13.54)	54.49 (13.88)	55.66 (10.97)	62.63 (10.41)	49.96 (14.24)	0.126	0.199
	HFnu-dBP	12.48 (10.75)	14.10 (10.82)	11.89 (5.66)	14.59 (8.75)	9.93 (7.52)	15.73 (12.98)	0.575	0.895
	LF/HF-dBP	6.13 (4.20)	6.94 (5.44)	5.76 (2.69)	5.91 (3.87)	11.09 (8.28)	6.29 (4.97)	0.129	0.763
**Systolic BP variability**	LFnu-sBP	38.48 (13.86)	46.41 (14.08)	46.10 (16.33)	46.85 (11.71)	56.91 (11.52)	43.10 (14.12)	0.114	0.299
	HFnu-sBP	15.43 (10.68)	17.58 (11.83)	14.89 (5.54)	16.77 (10.22)	9.44 (8.33)	20.98 (13.21)	0.094	0.220
	LF/HF-sBP	3.80 (2.43)	4.68 (4.41)	3.48 (1.43)	4.14 (2.80)	10.58 (7.30)^$ $^	3.33 (2.48)	< .001**	0.193
**Baroreflex sensitivity**	ms/mmHg	11.66 (9.58)	13.63 (8.83)	20.68 (16.40)^$^	13.17 (9.35)	19.10 (7.50)	10.47 (4.49)^$^	0.012*	0.070
**Baroreflex effectiveness index**		58.08 (15.49)	60.77 (18.71)	64.63 (32.27)	56.26 (16.39)	73.53 (18.61)	57.51(18.69)	0.150	0.401
**Active Stand**	nadir sBP	89.70 (16.66)	98.88 (26.60)	101.33 (17.63)	111.78 (29.45)	85.75 (28.50)	97.88 (25.96)	0.249	0.450
	drop sBP	19.80 (9.19)	11.44 (12.20)*	7.00 (6.50)	13.57 (17.13)	13.79 (8.00)	11.01 (12.54)	0.719	0.806
	drop > 20mmHg	5 (50%)	9 (18%)*	0 (0%)	2 (22%)	1 (12%)	6 (23%)	0.571	-
	30:15 ratio	1.14 (0.18)	1.20 (0.31)	1.37 (0.67)	1.28 (0.36)	1.15 (0.20)	1.15 (0.17)	0.355	0.521
	AUC baseline	259.3 (294.3)	109.9 (256.4)	29.95 (45.73)	274.8 (503.8)	82.26 (98.97)	79.72 (171.3)	0.191	0.977
**Valsalva**	minimum RR	478.9 (126.0)	522.4 (120.6)	469.8 (94.7)	585.0 (128.5)	488.9 (99.9)	523.2 (125.3)	0.246	0.591
	RR rebound	981.2 (213.1)	788.1 (210.3)*	880.5 (244.7)	749.9 (180.9)	748.8 (210.4)	792.5 (217.2)	0.640	0.705
	Peak I sBP	140.00 (17.33)	137.70 (23.76)	143.33 (13.84)	139.00 (24.11)	132.13 (30.45)	137.67 (24.15)	0.857	0.683
	Trough IIe sBP	101.78 (15.63)	108.62 (21.16)	108.50 (20.47)	114.67 (21.07)	102.38 (24.99)	108.46 (20.79)	0.711	0.705
	Peak III sBP	126.89 (12.66)	134.79 (24.06)	134.17 (20.46)	143.78 (24.60)	122.38 (30.02)	135.71 (22.27)	0.339	0.834
	Trough III sBP	118.44 (15.43)	126.38 (25.87)	123.50 (19.55)	135.00 (26.15)	112.75 (28.97)	128.42 (25.72)	0.336	0.811
	Peak IV sBP	148.33 (30.76)	143.47 (27.39)	152.00 (19.72)	147.89 (28.64)	136.50 (26.05)	142.00 (29.65)	0.718	0.511
	AUC (baseline)	233.2 (371.3)	115.2 (137.4)	107.9 (70.1)	134.22 (134.0)	68.0 (90.1)	125.6 (164.2)	0.749	0.638
	Time below (1^st^ to last)	15.83 (19.54)	11.60 (12.25)	12.28 (6.24)	12.07 (10.38)	7.28 (5.98)	12.70 (15.34)	0.760	0.441
	Time below (baseline)	15.18 (19.73)	9.68 (11.16)	8.24 (5.26)	11.22 (10.07)	6.14 (6.16)	10.65 (13.76)	0.752	0.818

Values expressed as mean (SD) or n (%)

* significant at 5% level

** significant at 1% level

$ $ significant post-hoc differences from all other subgroups

$ significant post-hoc differences between indicated groups

# two patients and one control participant were unable to complete Valsalva manoeuvre

There were no statistically significant differences in HR and BP between DSQ subgroups. Fukuda alone subjects had the lowest HR and Fukuda+2003+2011 the highest. Although not statistically significant, mean measures in the Fukuda+2003+2011 group are overall lower for all parameters except TPRI. Measures in the Fukuda alone group are higher compared to other subgroups, also with the exception of TPRI.

### Autonomic nervous system in response to standing and Valsalva

Neither mean BP nor nadir on standing was statistically or clinically significantly different and 30:15 ratio–an indicator of parasympathetic activity–was within normal parameters.

The CFS group has a statistically significantly longer Valsalva ratio compared to the control group; however, both groups had an abnormal ratio (<1.21) indicating dysautonomia. Low baseline blood pressure may help to explain these results.

Valsalva ratio was abnormal in all DSQ subgroups indicating dysautonomia. There are statistically significant between-group differences in Phases I and Iii, which may reflect parasympathetic activation by the baroreceptors[33].

### Cognitive performance

Results are detailed by domain tested and shown in Table 5.

**Table 5:** Cognitive testing by DSQ.

	all CFS	Fukuda	Fukuda	Fukuda +	Fukuda +	p value	p value
			+ 2003	2003	2003 +	(ANOVA)	(Kendall
			Clinical	Research	2011		tau-b)
	(n = 49/48)#	(n = 6)	(n = 9)	(n = 8)	(n = 26/25#)		
**Rey AVLT**							
Total recall trials 1–5	47.2 (8.8)	51.3 (6.3)	49.4 (11.7)	53.0 (4.47)^$^	43.7 (7.8)^$^	0.017*	0.001**
Forgetting (% retained from A6 on trial A7)	75.7 (21.2)	80.1(23.2)	80.3 (12.0)	84.2 (14.8)	70.5 (24.2)	0.322	0.112
**Forward Digit Span**							
Clinical measure	6.6 (1.4)	6.7 (1.2)	6.9 (1.5)	6.4 (1.2)	6.6 (1.5)	0.906	0.772
**Spatial span**							
Forward longest sequence	5.1 (1.3)	6.3 (0.8)^$^	5.4 (1.5)	5.6 (0.9)	4.5 (1.1)^$^	0.002**	<0.001**
Backward longest sequence	4.9 (1.2)	5.0 (0.9)	4.8 (1.6)	5.9 (0.6)	4.6 (1.2)	0.066	0.184
**VPT**							
Maximum number of targets	9.9 (2.3)	10.5 (1.2)	11.0 (2.0)@	11.4 (1.9)@	8.8 (2.2)@@	0.005**	0.003**
**DSST**							
Symbols per second	0.58 (0.16)*	0.7 (0.1)^$^	0.6 (0.1)	0.7 (0.1)	0.5 (0.2)^$^	0.016*	0.011*

Values are expressed as mean (SD)

* significant at 5% level

** significant at 1% level$ significant post-hoc differences between groups

@ significant post-hoc differences of these groups and @@ group

# One participant was unable to complete Spatial Span and VPT

### Memory: Verbal

There was a statistically significant inter-group difference in total recall over trials 1–5 on AVLT. Participants in the Fukuda+2003+2011 group underperformed (score 43.7) compared to the other groups–an indication of a less efficient verbal declarative memory–with those meeting a Fukuda+2003 Research ‘diagnosis’ performing best (53.0). The difference between these groups resulted in the only significant post-hoc test. Furthermore, the Fukuda+2003+2011 group also had the lowest percentage retention on trial A7 (70.5%) and the Fukuda+2003 Research group the highest (84.2%), but this was not statistically significant.

### Memory: Visuospatial

There are statistically significant differences across all measures on spatial span and VPT at least at trend level. Participants in the Fukuda+2003+2011 group had the lowest scores in forward spatial span and VPT compared to the other subgroups. For forward spatial span the difference to the Fukuda only group reached statistical significance in post hoc tests. For VPT the difference to the two Fukuda+2003 groups was significant. This suggests this group has a deficit in immediate spatial memory compared to the others[34, 35]. Although the Fukuda+2003+2011 group also has the lowest score in backward spatial span among the patient subgroups and there is a difference between the subgroups in this measure at statistical trend level (p = 0.066), this seems to be driven more by higher scores in the Fukuda+2003 Research group (M = 5.9) than by the poor performance in the Fukuda+2003+2011 group, but none of the post-hoc tests reached statistical significance.

These findings suggest that visuospatial working memory, which allows temporary retention and manipulation of information[36] (spatial span) and short-term visuospatial memory (VPT)[37], differs by diagnostic criteria and may be impaired in participants meeting Fukuda+2003+2011 criteria. There was an effect of group on the number of symbols recorded per second on the DSST, with the fewest symbols completed in the Fukuda+2003+2011 group, which was statistically different from the Fukuda only group in post-hoc tests.

## Discussion

CFS subjects reported significantly greater autonomic and cognitive impairment compared to matched, sedentary controls; however, findings did not show significant objective autonomic differences. There were significant differences in subjective cognitive and autonomic measures on DSQ categorisation, as well as clinical differences in objective assessment of autonomic and cognitive function. Possible interpretation of these results is discussed below.

### CFS versus controls

The lack of statistically significant objective difference in autonomic function in this cohort of CFS and control subjects differs from previous studies that have demonstrated a difference in autonomic function[19, 38] and may reflect a number of factors. Firstly, sedentary controls were selected for participation. Previously demonstrated autonomic dysfunction in CFS subjects compared to controls may have arisen secondary to inactivity and consequent deconditioning.

Secondly, this study excluded participants with a history of co-morbid depression. Autonomic dysfunction has been found in depression[39]. It is possible that autonomic dysfunction seen in other studies results from co-morbid depression and is not a primary feature of CFS. Consistent exclusion criteria across studies and research centres would enable better delineation of these two conditions and their presenting symptoms.

Finally, it is feasible that autonomic dysfunction experienced by CFS subjects, manifesting as HR and BP abnormalities, is intermittent. Although assessment using the TFM provides a continuous measure it is conducted over approximately 20 minutes implying that intermittent symptoms may not be captured.

### DSQ

Autonomic function measured using HR and BP variability assessed over a 10-minute rest suggests differences in the balance between sympathetic and parasympathetic autonomic function with Fukuda+2003 Research showing greater sympathetic activity and Fukuda+2003+2011 lower sympathetic modulation of cardiovascular activity.

LF/HF-sBP is highest in the Fukuda+2003 Research group suggesting a shift towards greater sympathetic activity in this group. It is lowest in the Fukuda+2003+2011 group and suggests lower sympathetic activity, further supported by a low LFnu-sBP indicative of low sympathetic modulation of sBP, and of a higher HFnu-sBP, suggesting greater parasympathetic modulation.

Although not statistically significant, this points towards the presence of different phenotypes across DSQ subgroups, suggesting that AD may differ by diagnostic criteria when considered at a clinical level.

The differences observed between DSQ subgroups may be a reflection of the *additive* effect in diagnostic criteria. The absence of autonomic symptoms in the Fukuda criteria implies a different, less severe, disease phenotype with fewer features of AD. In contrast, symptoms of AD are present in the 2003 criteria and include ataxia, muscle weakness and OI. Further still, the 2011 criteria have a requirement for a greater symptom burden, which can include widespread migratory pain and hyperalgesia.

The possibility that current criteria include symptoms that are not primary features of CFS, specifically in relation to the Canadian 2011 criteria which encompasses a broad spectrum of symptoms across body systems, and confounds clinical presentation and research, must be considered. The number of symptoms across many physiological symptoms included in the Canadian 2011 criteria may mean that, rather than diagnosing a more severe CFS phenotype, these criteria capture both CFS and other co-morbidities with non-specific symptoms. This may not only affect management and subsequent prognosis, but also give rise to an inaccurate and confused picture of which condition (or conditions) is being researched and serve to exacerbate the stigma associated with the condition.

Furthermore, it is possible that the inclusion of more widespread pain with or without hyperalgesia in the 2011 criteria may be a confounding symptom–particularly in view of the association between pain and both autonomic dysfunction and cognitive impairment (attention, psychomotor speed, verbal and working memory)[40–42].

There was only a statistical trend for a monotonic relationship between DSQ subgroups and self-reported cognitive impairment with COGFAIL. Fukuda+2003+2011 subjects reported higher scores than all other subgroups, pointing towards greater impairment in this group.

Comparison of objective measures between DSQ diagnostic subgroups revealed statistically significant between-group differences in verbal memory–as assessed using the AVLT total score, visuospatial memory and psychomotor speed, with the Fukuda+2003+2011 subgroup consistently showing greater impairment, in particular than the Fukuda alone group. These findings mirror subjectively higher scores of cognitive impairment on COGFAIL, greater fatigue on FIS and more autonomic symptoms on COMPASS. Fukuda alone and Fukuda+2003 Clinical appeared to perform better.

Given the findings of the ANOVAs and the patterns of post-hoc results, there appears to be an additive effect in the DSQ criteria where more symptoms result in greater symptom burden and disease severity and points towards a subgroup of patients with greater functional impairment. The results of the monotonic trend tests (Kendall tau-b) often support this notion. These findings contribute to a picture of potentially clinically significant and distinct phenotypic differences between DSQ subgroups and the possibility of CFS as a disease spectrum.

One of the study strengths was that it recruited a highly-motivated cohort of CFS subjects who completed a comprehensive series of investigations enabling, for the first time, in-depth observation of the potential phenotypic differences between DSQ subgroups. There are a number of limitations to acknowledge including the small number of controls and small sample sizes in DSQ subgroups, which means the study is underpowered. In particular the comparisons between the full patient sample and the small control sample are likely to suffer from problems with variance inhomogeneity. Conducting clinical research in this group of patients presents challenges in recruiting adequate numbers and this study would have benefitted from additional control subjects to increase statistical validity. The study results point towards the possibility of a spectrum of disease phenotypes as shown across diagnostic criteria. These phenotypes appear to be quantitative in nature, i.e. of symptom/disease severity, however a small study population of CFS subjects well enough to attend several appointments and therefore likely to represent milder disease severity, mean this is difficult to characterise. Future studies would benefit from exploring the possibility that clusters of participants represent different disease phenotypes to better explore whether they represent discrete, qualitative traits. This study is observational and therefore disease causality cannot be determined.

## Conclusions

Findings of no significant differences in objective autonomic parameters between CFS and control groups raises questions about the role of co-morbid depression and sedentary controls and strongly indicates that more consistent inclusion and exclusion criteria across studies is key to furthering understanding of CFS pathophysiology.

This is the first study investigating objective differences in autonomic and cognitive features across DSQ subgroups. It suggests that CFS–as classified using current diagnostic criteria–may constitute a disease spectrum, with different severities. This has potentially significant repercussions for further understanding of the underlying aetiopathogenesis of this debilitating condition, contributing to the sometimes conflicting and mixed results seen across studies, and implications for response to clinical management.

This study suggests that there are differences in objective autonomic parameters across groups of CFS subjects. Damage to the autonomic nervous system appears to take the form of an initial sympathetic over-modulation followed, in more severe disease, by sympathetic underactivity and increased parasympathetic modulation, as seen with subjects meeting the Fukuda+2003 Research and Fukuda+2003+2011 criteria respectively. This supports the theory that abnormalities in the autonomic nervous system are a potentially important feature of CFS and hold promise for better understanding the underlying pathophysiology of this condition.

This study highlights the need for international consensus with regard to diagnosis. The recent development of the Institute of Medicine criteria will go some way to achieving this but it is vital that there is consistency in diagnostic approach when performing research in this condition where novel therapies are so urgently needed.

## Supporting information

S1 File Dataset. (XLSX)
